# Chicago Medical Society

**Published:** 1884-04

**Authors:** 


Adjourned. L. H. M.

---

CHICAGO MEDICAL SOCIETY.

The following embodies the scientific proceedings of the meeting held at the Pacific on the evening of February 18, 1884.

The first paper was read by Dr. Wm. L. Axford, who gave a report of "A Case of Ventral Hernia," occurring in a male, and exhibited the large pathological specimen.

The following in brief is the report substantially as that given by the author:

It is my privilege to present before the society this evening a pathological specimen, removed post-mortem, from a very interesting case, which for a long period of time had been the subject of considerable doubt to a number of physicians. The history of the case is as follows:

F. B., aged 56, weight 340 pounds, an enormously large and fleshy man; had been troubled for the past twenty years by a tumor, situated above and to the left of the umbilicus. Did not know any cause for its appearance (having never received any injnry along the linea alba), which was at first that of a slowly growing tumor. He wore a pad for a number of years, and in this way was able to keep the mass within bounds, but during the past five years it became uncontrollable and had been a constant source of trouble, dating back to a very severe attack of constipation, followed by severe vomiting; in fact, so near as I can learn, at that time he had all the symptoms of a strangulated hernia. Two years before death another strangulation occurred, and at that time it was my good fortune to see him. In each instance the symptoms of strangulation seemed to have subsided under the use of opiates, with rest in bed. In the intervals the patient had weekly attacks of what was described as a hardening of the mass; at such times it would increase to an enormous size, include the umbilicus in the swelling, and become extremely painful, but he did not vomit. He would go immediately to bed, and after a day or two of rest it would subside.

When I first saw the case, at the time it was strangulated for the second time, the tumor was about the size of an adult head, hard and tense, but resilient to the touch.

Percussion gave dullness over the upper and left part of the mass, while over the lower two-thirds the percussion note was tympanitic.

A number of diagnoses had been made by a variety of physicians, such as tumors of various kinds, particularly fatty tumor, hernia, hernia with fatty tumor, etc., etc. The physician who first treated the case evidently took it for a hernia, as he treated it by a pad, and controlled it for a long time. For my own part I believed it to be a ventral hernia containing intestine and omentum, thinking that the dullness over the left and upper part could be well accounted for by supposing that so large and obese a man would naturally have a thick, fatty omentum, which, from its anatomical relations, could easily occupy the left and upper portion of the hernial sac.

Two years later I received an invitation to assist at the autopsy, the patient having died with symptoms similar to those of the two previous occasions.

I now have the pleasure to present to you the sac of the hernia. No omentum was found in the sac; it contained small intestines alone.

In the discussion considerable contrariety of opinon was manifested regarding the use of a rubber bandage applied in treating this variety of hernia, as well as the utility of it in femoral, strangulated femoral, and other forms of rupture that occur in both sexes. Its advantage was affirmed by some and denied by others. It was also argued by one speaker that he personally had met a case of strangulated femoral hernia that recovered without sloughing or an operation, and that so far as he knew little or no treatment had been pursued, which result he admitted was a surprise to him, for when he was called to see the patient the latter was suffering acutely from well marked symptoms of strangulation, yet within a few days the man recovered and resumed his duties attending a garden of which he was owner.

The second paper was read by Dr. L. A. Harcourt, who reported three cases of arrested fœtal development. In the most interesting case there was entire absence of the abdominal walls and the umbilical cord. A synopsis of the cases reported is hereby appended.

CASE I.—On the 4th of March, 1881, I was called to see Mrs. C., living on S. May St., in labor with her fifth child. Some months before, she had been threatened with an abortion, having had pain and hæmorrhage for several days; but rest and anodynes averted the calamity. I attended her at the time, knew she was pregnant, but did not look for her confinement so soon.

On examination in the present case, found the os uteri about two-thirds dilated, the membranes protruding into the vagina, but only a membranous tumor could be felt by the finger. Thinking there might be a breech presentation, and that instrumental aid might be necessary in the delivery of the head, I went for my forceps, a couple of blocks away, requesting the lady in the meantime to go to bed. Returning in a few moments, I found her in severe pain, the membranes protruding from the vulva, and as the pain subsided, I passed my finger beyond the tumor, which was a spina bifida, and distinctly felt the fœtal head. The os uteri being fully dilated, I ruptured the membranes at the next pain, and almost in an instant, the child was swept into the world with extraordinary force. I felt the warm blood gush on to my hand, and feared I had a case of post-partum hæmorrhage to deal with. A glance at the woman's face, however, banished my fear, and left me to look for some other explanation of the hæmorrhage. The blood came from another source, however. The child and placenta came into the world simultaneously. Looking at the child, which was alive, I saw its face grow white, and a second look revealed the fact that its abdominal parietes were wanting. The anterior and part of the lateral walls of the abdomen were absent, from a little above the os pubis to a little below the cuneiform cartilage, leaving the abdominal viscera expos.d. The intestines of course protruded, and the bladder, kidneys, stomach, and other organs could be distinctly seen and felt. The integument of the abdomen above, below, and laterally degenerated into membrane, and a membranous fringe from three-fourths of an inch to an inch and a half in width, resembling that attached to the placenta, and from which something had been irregularly torn, encircled the opening.

The lower extremities formed an obtuse angle with the body, the hips projecting to one side, as if the limbs of one fœtus had been engrafted obliquely upon the trunk of another. There was spinal curvature in the lumbar region. Altogether, it was something of a monstrosity, the sex of which I have forgotten. An examination of the child, the placenta, the vagina of the woman, and the bed, failed to discover the slightest trace of an umbilical cord. My impression is, that the membranes were attached directly to the child's abdomen, and were torn from it by the child's expulsion. The child lived only a few moments. The mother made a good recovery. She was not permitted to see the child until it was dressed, but knew there was something wrong, as no cord had been tied. She told me afterwards, in explanation of the abnormal condition of things, that, before she was threatened with an abortion, she had seen a man, who had fallen from a scaffold, whose abdomen was so torn that the intestines protruded from the rent, and who died in a few hours. The shock she received at the time, she thought caused an arrest of fœtal development, giving rise to the condition mentioned.

When called to the case, I was in attendance upon another lady whose labor was tedious, and to whom I was anxious to return; hence my examination of the fœtus before leaving was not as thorough as it ought to have been. On my return the child was dressed, and the afterbirth destroyed.

In the interest of science, I tried to get the child, offering to charge nothing for my services, if they would give it to me; but while the father seemed to appreciate my motives, he said, the mother would not listen to the proposition under any circumstances. Had I secured it, I would have written up the case long ago, and exhibited the fœtus to the society.

CASE II.—Some twelve years I was invited by Dr. Tousley, of Weyanwega, Wis., to see what he called a headless child recently born. I went with him and found the statement only partially true. The essential part of the head, the brain, was there, a part of its osseous covering alone being absent. It was a case of imperfect or arrested development of the cranial vault, a part of the frontal, temporal, parietal and occipital bones being wanting. They were absent from a line beginning a little above the supraorbital arch, passing backwards, arching slightly over each ear, aud passing through the occipital proturbance. The brain and its coverings were intact, the longitudinal fissure well marked, giving rise to two yielding tumors which the doctor at first mistook for the breech. The face was broad and flat, the nose flattened, the eyes small, round and protruding, probably from pressure upon the brain during labor. Every feature was distorted and repulsive in the extreme, the countenance resembling in some measure that of a hog.

The mother was a German, and she too had an explanation to offer. It was that some time after conception she came suddenly where hogs were being butchered, and saw a number of them decapitated. She was startled; hence, as she thought, her misfortune.

CASE III —While in Clinton, Wis., I was called to see a man who had fallen from the rafters of a house a story and a half high, striking against the joists and sleepers in his descent, and sustaining some injury about the back of the neck and between the shoulders. I removed his shirt to examine his injuries, and found an abrasion of the integument extending from the occiput to the second or third dorsal vertebra. The injury, though slight, looked bad at the time, the shoulders and upper part of the back being more or less spattered with blood. While stripped, his wife came to the place, and seeing her husband bleeding, became very much excited and alarmed. She was then in the third month of pregnancy. About four months later she prematurely gave birth to a child, on whose neck and between whose shoulders was a mark, the exact counterpart of the injury sustained by its father some months before. A triangular space extending from the occiput downwards between the shoulders was covered by a reddish membrane, presenting, in miniature, the appearance of the father's neck when seen by the mother. Reddish spots were scattered about the back and shoulders, corresponding to the blood stains on the father.

These three cases came under my personal observation, and the facts are truthfully given. Whether the maternal impressions and the subsequent morbid developments stand in the relation of cause and effect, or were mere coincidences, it is not my province to determine; but they seem to confirm the belief entertained by many, that Jacob knew whereof he bargained when he agreed with Laban to take as hire all the brown lambs and speckled goats and cattle born after a given date.

Discussion of moles, or maternal impressions, or mother's marks, was participated in by a number of the members.

Dr. A. Lagorio recited briefly the main points concerning a case he saw last April, although the woman who had been confined was attended by a midwife. A babe was born having double hare lip extending through the alæ of the nose. The nose was flattened; there was a cleft in the palate; the eyes were closed, and the lids could not be separated; there was no rotundity of the eyeballs. The child lived fifteen or twenty days, dying from inanition. At the autopsy there was discovered an entire absence of the eyes. The mother was 40 years old; an Italian. She had undergone during the period of gestation great hardships, such as scrubbing, using a pick-axe to remove stumps and roots; she also carried weights on her head, such as coal sacks. The poor woman supposed the midwife to be a witch, and it was her belief that her babe had been marked by the midwife just before its birth.

He believed there were no cases on record in this country where a child had been born without eyes, or where the lids could not be separated. In Vienna three cases have been reported where the eyes were missing, or where the eyelids were closed at birth. In one of these cases the lids were separated and the eyes opened, and subsequently the child perceived objects.

Dr. S. J. Avery gave a history of a case he had seen that may properly be regarded as coming under the subject discussed in the paper. Being called to attend a female patient about to be confined he met with a breech presentation. All went on very well until delivery of the shoulders, which act, however, was accomplished, but with more than ordinary difficulty. In the delivery of the head, he discovered that this was hydrocephalic, and measured, he thought, eight or nine inches antero-posteriorly. In the delivery cephalotomy was performed. The mother recovered with no serious trouble or cellulitis.

Dr. J. G. Kiernan gave an abstract of a number of cases that had been reported in different journals and other literature upon this subject, purporting to be derived from embryologists. Some of the cases cited were very extraordinary. Men skilled in embryology have little doubt but that a sudden and violent mental shock may arrest development in a fœtus.

The Secretary knew that Dr. Harcourt's actual experience had been reported in the paper, and he had requested it might be presented to the society.

Adjourned. L. H. M.

---

DECISION UPON AN ANATOMY ACT.—The law of Maryland makes it a criminal offence to take bodies from the ground after burial for dissecting purposes, unless these bodies are buried in potter's field. "Last spring," says the *Maryland Medical Journal*, "three men were arrested for having in their possession bodies obtained from Bay View, and being tried before Judge Fowler, at Towsontown, were convicted. The counsel for the defense maintained that the Alms-house burying ground was a potter's field, and hence was excepted by law, but Judge Fowler did not entertain this opinion, and the prisoners were declared to be guilty. This decision was appealed from, and after several trials the question was finally settled by Chief Judge Yellott on Friday last, who said that 'any place where unclaimed paupers or strangers were buried was a potter's field,' and the prisoners were acquitted, after a confinement in jail of eleven months in one case."—*Medical Record*, March 8, 1884.

